# Daily and event‐driven pre‐exposure prophylaxis for men who have sex with men in Belgium: results of a prospective cohort measuring adherence, sexual behaviour and STI incidence

**DOI:** 10.1002/jia2.25407

**Published:** 2019-10-30

**Authors:** Bea Vuylsteke, Thijs Reyniers, Irith De Baetselier, Christiana Nöstlinger, Tania Crucitti, Jozefien Buyze, Chris Kenyon, Kristien Wouters, Marie Laga

**Affiliations:** ^1^ Department of Public Health Institute of Tropical Medicine Antwerp Belgium; ^2^ Department of Clinical Sciences Institute of Tropical Medicine Antwerp Belgium

**Keywords:** PrEP, HIV prevention, MSM, Belgium, adherence, STI, event‐driven

## Abstract

**Introduction:**

Pre‐Exposure Prophylaxis (PrEP) is highly effective in reducing the risk for HIV infection among men who have sex with men (MSM) and may have an important impact in slowing down the HIV epidemic. Concerns remain however about low adherence, increased risk behaviour and reduced condom use when using PrEP. The aim of this study was to assess these factors prospectively among MSM using daily and event‐driven PrEP in Belgium.

**Methods:**

An open‐label prospective cohort study was conducted from October 2017 to May 2018 at the Institute of Tropical Medicine, in Antwerp, Belgium. At enrolment, MSM at high risk for HIV chose between daily or event‐driven PrEP. They were allowed to switch regimens or stop taking PrEP at each of their tri‐monthly visits. Data were collected on an electronic case report form, web‐based diary and self‐administered questionnaire. Screening for HIV and other Sexually Transmitted Infections (STIs) was also performed.

**Results:**

Two hundred MSM were followed up for a total duration of 318 person‐years. At month 18, 75.4% of the participants were on daily and 24.6% were on event‐driven PrEP. The mean proportion of covered sex acts by PrEP for the complete follow‐up period was 91.5% for all participants, 96.5% for daily and 67.0% for event‐driven PrEP use. The number of casual and anonymous sex partners was significantly higher for daily users, as compared with event‐driven users, but did not change over time. In contrast, the mean proportion of condomless receptive anal intercourse with casual and anonymous partners increased significantly during follow‐up, for both daily and event‐driven use (*p* < 0.0001 for all 4 trends). No new HIV infection was diagnosed during follow‐up. The incidence of bacterial STIs was 75.4 per 100 person‐years (95% CI 63.8 to 89.1). We did not detect a significant change in *N. gonorrhoeae*/*C. trachomatis* incidence over time. The incidence of hepatitis C was 2.9 per 100 person‐years.

**Conclusions:**

PrEP is an effective and well adopted HIV prevention tool for MSM in Belgium. Participants adapted daily and event‐driven regimens to their own needs and were able to adapt their PrEP adherence to risk exposure.

## 1 Introduction

Pre‐Exposure Prophylaxis (PrEP) using daily oral tenofovir disoproxil fumarate and emtricitabine (TDF/FTC) is highly effective in reducing the risk for HIV infection among men who have sex with men (MSM) 1, 2, 3. In addition, a randomized controlled trial among MSM in France and Canada demonstrated a 86% efficacy of a non‐daily, event‐driven regimen 4.

These results underscore the potential impact of including PrEP, daily or event‐driven, into a powerful HIV prevention package for the most‐at‐risk MSM. Concerns remain, however, about “real‐life” use of PrEP, including low adherence, increased risk behaviour and reduced condom use, upsurge in sexually transmitted infections (STIs), and long‐term drug toxicity.

Adherence is critical for the effectiveness of PrEP and poor adherence may explain incident HIV infections while on PrEP 5. Studies from other health domains showed that adherence for primary prevention may be more difficult to achieve than for secondary prevention 6. In addition, PrEP adherence must be understood within the context of variable risk for HIV infection and use of other HIV prevention methods 7, 8. In contrast to anti‐retroviral treatment which need lifelong sustained adherence by people living with HIV, PrEP can be taken temporarily during episodes of anticipated increased risk 9. Non‐daily regimens also have the advantage of requiring fewer tablets, thus reducing potential side‐effects and cost 10, 11. However, adherence to these regimens should be optimal, as they are less forgiving of missed doses 12. Although some studies report a lower adherence to non‐daily regimens, it remains unclear how good adherence is in a real life situation, when users chose between daily and event‐driven PrEP according to their preference 13.

Another concern raised in relation to PrEP refers to “risk compensation,” or a shift towards more risky sexual behaviour triggered by perceptions of decreased HIV risk 14. A systematic review and meta‐analysis of seventeen open‐label studies found that PrEP among MSM was associated with a decrease in overall condom use but no increase in HIV 15. However, these changes in sexual behaviour may increase the chances of acquiring STIs other than HIV 16. We still lack data on the incidence of STIs and their trends in MSM while on daily and event‐driven PrEP.

Belgium is among the countries in the European Union reporting a high HIV incidence, with 7.9 new HIV infections per 100,000 inhabitants in 2017 17, 18. A recent study suggests that ongoing clustered transmission in Belgium is almost exclusively MSM driven 19. At the time of this study PrEP was not yet approved or reimbursed. Since 1 June 2017, Truvada can be prescribed and reimbursed as prophylactic medication for people who are at increased risk of HIV acquisition in Belgium.

The aim of this study was to assess adherence, sexual risk behaviour, condom use and STI incidence among daily and event‐driven PrEP users in a prospective cohort of Men and Transgender Women who have sex with Men (MTSM) at high risk for HIV in Belgium.

## 2 Methods

### 2.1 Study design, setting and participants

Be‐PreP‐ared was a single‐site, open‐label prospective cohort study (EudraCT 2015‐000054‐37) conducted at the Institute of Tropical Medicine, in Antwerp, Belgium.

We enrolled 200 HIV negative MTSM, aged 18 years or more, who were at high risk of HIV acquisition. Potential participants were recruited through study advertisement at websites and various community‐based gay and sexual health organisations. High risk was defined as reporting at least one of the following in the previous six months: unprotected anal sex with a casual partner with unknown or HIV positive status, a STI diagnosis and post‐exposure prophylaxis. Exclusion criteria included HIV infection and contraindications for the study drug, as published previously 20. We calculated a sample size of 200 to estimate the proportion of participants who were non‐adherent with a precision of 7% if the proportion of non‐adherence was 50%.

### 2.2 Study procedures and data collection

Detailed study procedures have been published before 20. Eligible participants provided written informed consent and were invited to an enrolment visit, a follow‐up visit after one month, and then 3‐monthly visits until 18 months. The first enrolment took place on 7 October 2015 and last enrolment on 12 December 2016. The study follow‐up period was extended until the end of January 2018 for those participants rolling out before 1 November 2017. The aim of the extension was to cover the window period between the end of the study and the anticipated availability of PrEP in Belgium. Data collection ended on 4 May 2018.

TDF 245 mg/FTC 200 mg was used as PrEP in this study. Participants received detailed information and counselling about the two different PrEP dosing regimens. They then choose between daily PrEP (one pill every 24 ± 2 hours) and event‐driven PrEP (a starting dose two to 24 hours before anticipated sexual contacts, and then continuing with the daily regimen until two days after the sexual active episode). The starting dose normally involves two pills unless the most recent dose was taken between one and six days before the starting dose. Participants could stop taking PrEP, or switch regimens at each visit.

Study procedures included a medical history and examination, sexual health counselling, and blood samples for HIV, serum creatinine and syphilis. HSV‐2 and hepatitis B virus (HBV) serology were performed at screening and at month 18. We performed serology for hepatitis C (HCV) initially at baseline and at month 18. After discovering two new HCV infections at month 18, we wrote an amendment to extend HCV serology every six months from 23 May 2017 on. Participants self‐collected urine and anorectal swabs and a study nurse took pharyngeal swabs for STI testing. We recorded all clinical, laboratory and adherence data on a standardized electronic case report form. At enrolment and at each follow‐up visit, participants completed a detailed electronic questionnaire on sexual behaviour from the previous three months, including number of partners, type of sexual contact (insertive or receptive) and condom use per type of partner (steady, casual, anonymous). Casual partners were defined as non‐steady, non‐anonymous partners with whom the study participant occasionally or regularly had sex.

Participants also completed a personal web‐based diary with information on daily pill intake and, if sexual activity took place, the HIV exposure predefined category (low, medium or high). The exposure category was suggested by an algorithm, taking into account type of sexual contact, condom use, type of sexual partner (anonymous, casual or steady), HIV serostatus of the partner and viral load if the partner was HIV positive. High HIV exposure was defined as condomless anal intercourse with a new or occasional partner who is HIV positive or of unknown status, or with a HIV positive steady partner with detectable viral load. Low HIV exposure was defined as consistent condom use during the whole “sex day,” or condomless anal intercourse with a steady partner who is HIV negative or HIV positive with undetectable viral load. Medium exposure was defined on sex‐days which did not correspond to “high” neither to “low” exposure.

### 2.3 Laboratory procedures

All laboratory procedures were carried out in the Central Laboratory for Clinical Biology or the HIV/STI Reference Laboratory of the Institute of Tropical Medicine, Antwerp, Belgium. HIV was tested using an on‐site point‐of‐care test (Alere HIV Combo, Alere Inc., Waltham, MA) confirming HIV negativity by two HIV fourth generation enzyme‐linked immunosorbent assays including HIV antigen testing according to the algorithm of the ITM 20. Syphilis was tested through Rapid Plasma Reagin (RPR, Macro‐Vue, Becton Dickinson BD Microbiology Systems, Maryland, USA) and Treponema Pallidum Assay/ Treponema Pallidum passive particle agglutination assay (TPA, Vitros 5600 Ortho‐Clinical Diagnostics, Rochester, NY)/SERODIA‐TPPA, Fujirebio Inc, Tokyo, Japan). In addition, real‐time PCR was used to detect *Neisseria gonorrhoeae* (NG), *Chlamydia trachomatis* (CT), *Mycoplasma genitalium* (MG) and *Trichomonas vaginalis* (TV) on urine, pharyngeal and anorectal samples according to previously published assays 20. Samples positive for CT were further tested to distinct L‐serovars from non‐L serovars using a previously published real‐time PCR 21. HSV‐2 antibody testing was done by the Kalon HSV‐2 IgG ELISA (Kalon Biological Ltd., United Kingdom). In case a sample was positive on month 18, a look back approach was applied and previous samples were tested until the most recent negative sample. We tested for HCV antibodies, HBsAg, HBsAb, HBcIg and HBcIgM, AST/ALT and creatinine with Vitros 5600 (Ortho‐Clinical Diagnostics, Rochester, NY). If a six‐monthly sample was positive for HCV, we also looked back at the previous sample to narrow down the time of infection.

### 2.4 Outcomes and definitions

#### 2.4.1 Adherence (based on diary data)

Adherence was estimated by the proportion of anal sex acts covered by PrEP. The proportion of anal sex acts covered by PrEP was calculated as the proportion of “sex‐days” (i.e. days when anal intercourse with one or more men occurred as denominator) for which PrEP was correctly taken (numerator). A correct intake of PrEP involved a correct dose of PrEP before, during and after the days on which sexual intercourse took place. A correct dose before involved at least two pills taken on days X (i.e. a sex day) or X‐1 (i.e. the day before); or at least one pill on X or X‐1 if a pill was taken between day X‐6 and X‐1. The last situation occurred when a person was on daily PrEP, or if there was less than one week between two episodes of event‐driven PrEP. A correct dose during and after included at least one pill on days X, X + 1 and X + 2.

All information on daily sexual activity and pill intake was extracted from participants’ diaries.

#### 2.4.2 Sexually transmitted infections

Participants were considered infected with NG if they tested positive for NG in one of the three biological sites (anorectal, pharynx or urine). The same was done for CT, MG and TV. A diagnosis of syphilis was defined as a positive RPR test with a titre of at least 4 together with a positive TPA or TP‐PA test. As for HSV‐2, a grey zone ratio of 0.9 up to 1.1 was coded as not interpretable.

### 2.5 Statistical analysis

The study statistician performed all statistical analyses using SAS 9.4 (SAS Institute Inc., Cary, NC, USA) and R 3.5.0 (R Foundation for Statistical Computing, Vienna, Austria, https://www.R-project.org/.

The analyses of adherence and sexual behaviour characteristics were done by regimen used since the last scheduled visit. In the latter case, if regimens were switched since last scheduled visits, participants were assigned to the regimen he/she took the longest time during this episode. To compare adverse events and STI incidence between the regimens, we took the regimen which was used the longest over the full follow‐up.

The proportion of covered sex‐days and the proportion of condomless receptive anal intercourse was estimated using a binomial Generalized Linear Model. The number of partners was estimated using a Poisson regression model.

The global incidence rate of STI was calculated with censuring after a positive test. To assess trends in the incidence rate of NG/CT over time, we calculated the number of times a participant had a positive NG or CT test result. A mixed effects Poisson regression model was fitted with visit as a categorical covariate, a random intercept per subject and log (follow‐up time) as offset. After each positive lab result, the participant was considered not at risk for 14 days to take the treatment period into account.

### 2.6 Ethical clearance

Ethical approval was provided by the Institutional Review Board of the Institute of Tropical Medicine Antwerp and the Ethics Committee of the Antwerp University Hospital.

## 3 Results

### 3.1 General characteristics of the study cohort and choice of PrEP regimens

A total of 197 men and three transgender women participated in the study. A detailed description of the socio‐demographic characteristics of the study participants at baseline has previously been published 22. Briefly, their median age was 38 years (min 22, max 70) and they were predominantly white (89.0%), highly educated (78.5%) and employed (78.5%). After 18 months of follow‐up, 89.5% of the 200 participants were still on PrEP. Extension of study participation was taken up by 99 participants. The total follow‐up time was 318 person‐years.

**Figure 1:**
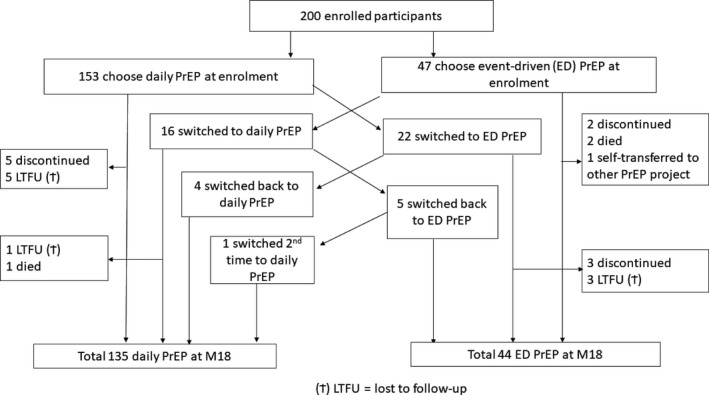
Flow diagram of study participants and PrEP regimen switching patterns.

Figure 1 shows details of the regimen initial choices and switches, discontinuation and lost to active follow‐up until M18.

Thirty‐eight participants (19%) switched regimens at least once. At M18, 135 participants were on daily (75.4% of those still on PrEP) and 44 were on event‐driven (24.6% of those still on PrEP) regimen. The main reasons for switching to the event‐driven regimen (in total 27 switches) included: change in sexual relationship (n = 12) or less sex (n = 4), too many pills to take (n = 6) and illness (n = 4). The main reasons for switching to daily (total 21 switches) included: too difficult to remember to take the pills (n = 15), change in sexual relationship (n = 5) or more frequent sex (n = 2), and convenience or easier to take (n = 3).

During their follow‐up, 64 participants interrupted their regimen temporarily, with a total of 95 interruption episodes and a median duration of 14 days (min 1 day to max 169 days). The main reasons for interruption included illness (n = 39), travel (n = 15), running out of pills (n = 12) and side effects of TDF/FTC (n = 11).

Overall, 128 participants (96 in daily and 32 in event‐driven regimen) reported 238 adverse events possibly, probably or definitely related to the study drug. Most were gastrointestinal such as flatulence, diarrhoea, nausea and gastric discomfort (76 under daily and 23 under event‐driven regimen).

The proportion of participants who expressed the intention to use PrEP in the future was 96% at their last study visit.

### 3.2 Adherence

One participant did not use the diary. The other participants filled in the diary for 95.5% of all follow‐up days.

**Figure 2:**
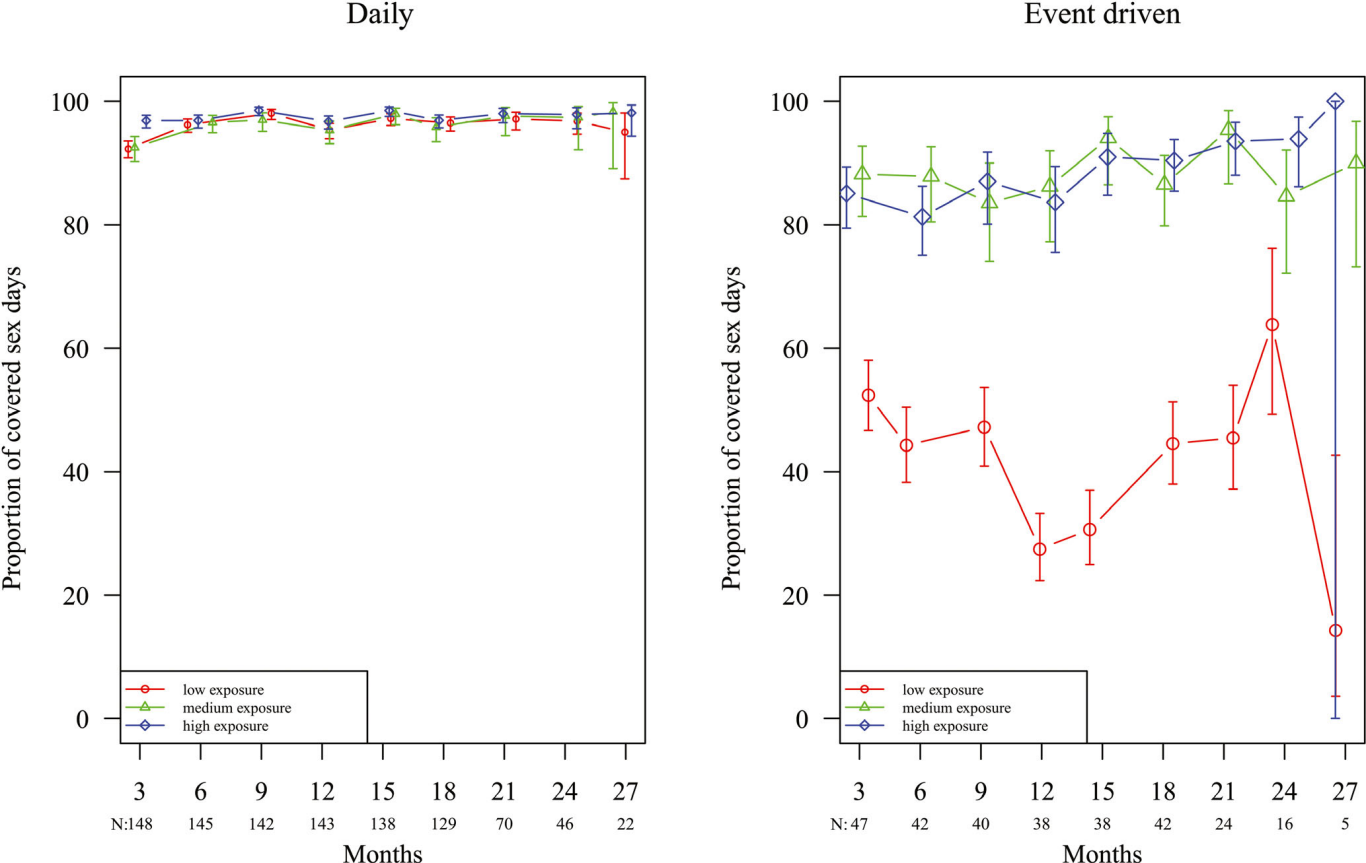
Proportion of covered sex days according to PrEP regimen.

The mean proportion of covered sex‐days for the complete follow‐up period was 91.5% (95% CI 91.1 to 91.8) for all participants. This proportion was 97.5% (95% CI 97.1 to 97.8) and 95.9% (95% CI 95.4 to 96.3) for daily PrEP and 87.7 % (95% CI 85.7 to 89.5) and 42.1 % (95% CI 39.8 to 44.5) for event‐driven PrEP), during days with high and low exposure respectively (mixed effects logistic regression model, all *p* < 0.0001). Figure 2 shows the evolution over time.

### 3.3 Sexual risk behaviour

**Figure 3:**
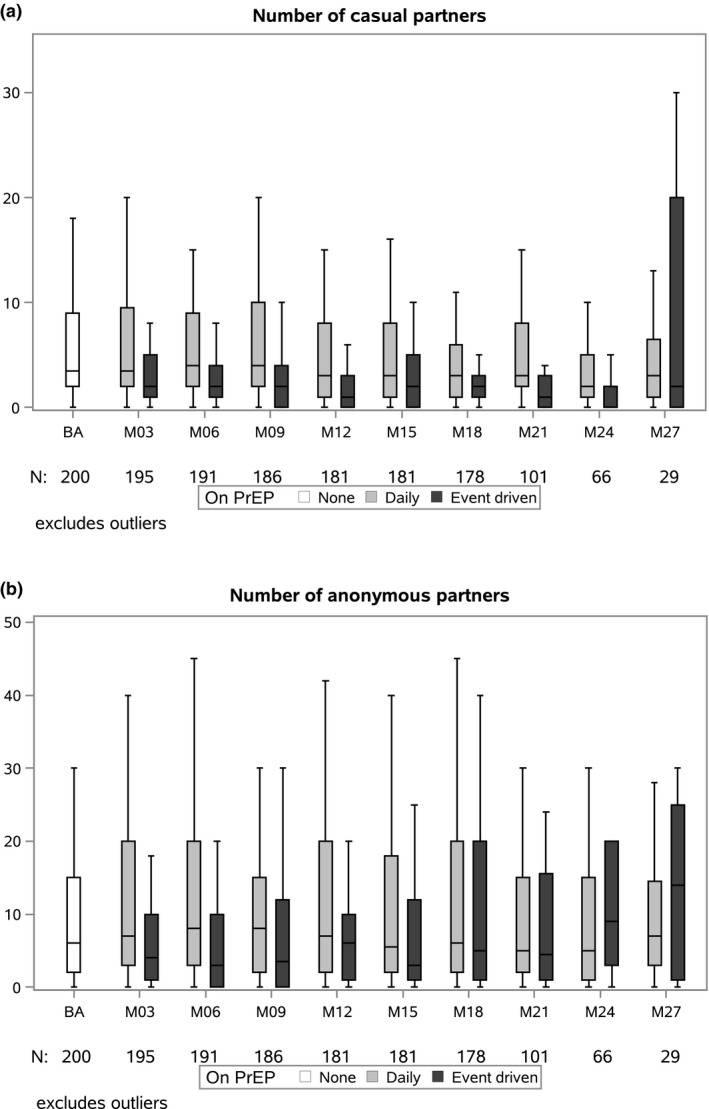
Summary boxplots of the number of casual (a) and anonymous (b) partners in last three months, per drug regimen.

Figure 3 shows the evolution of the number of casual and anonymous sex partners over time. This number was significantly higher for daily, as compared to event‐driven PrEP users (joint test of group coefficient and the interaction, *p* = 0.044), but did not change significantly over the study period in both groups (Poisson GEE).

**Figure 4:**
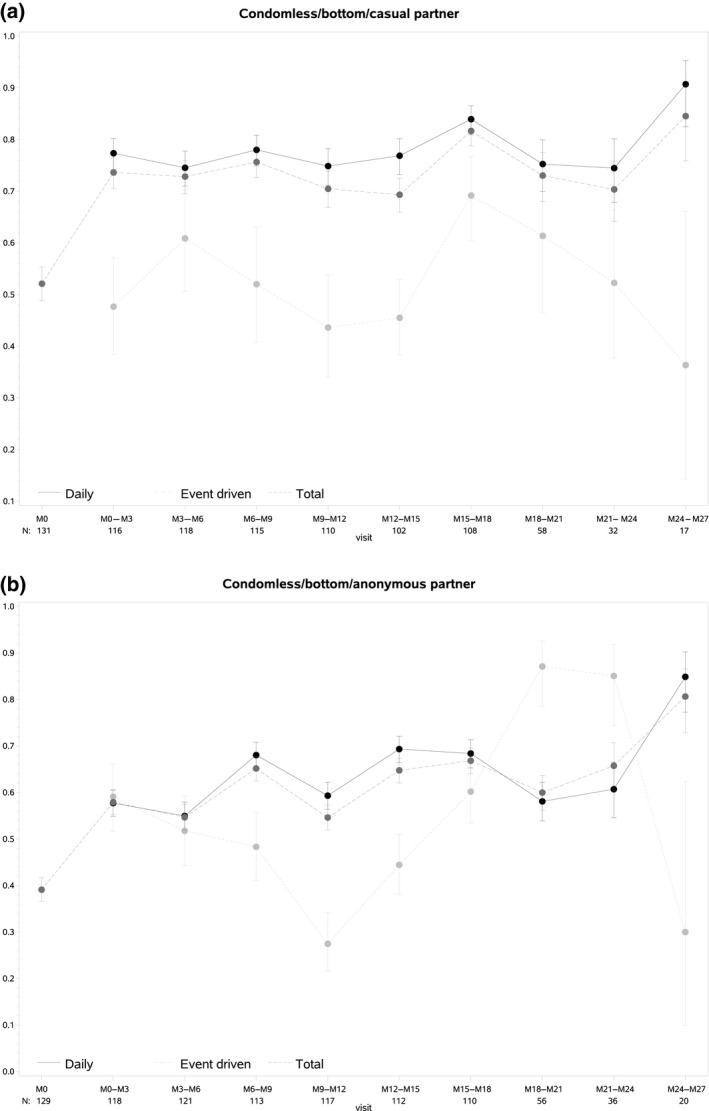
Proportion of condomless receptive intercourse with casual (a) and anonymous (b) partners over time, per PrEP regimen.

The mean proportion of condomless receptive anal intercourse with casual and anonymous partners increased significantly during follow‐up, for both daily and event‐driven use (mixed effects logistic regression model, *p* < 0.0001 for all four trends) (Figure 4). Participants’ own perception of changing condom‐use while on PrEP was assessed in the questionnaire. The proportion answering they use less condoms since starting PrEP was 44.3% on M3, 53.1% on M12 and 61.0% on M18.

### 3.4 HIV/STI incidence

During the 318 person‐years of follow‐up no new HIV infections were diagnosed. Table 1 summarises incidence rates of the other STIs assessed. The incidence of bacterial STIs (syphilis or NG or CT or MG) was 75.4 per 100 person‐years (95% CI 63.8 to 89.1). A total of nine new hepatitis C infections were reported during follow‐up, resulting in an incidence rate of 2.9 per 100 person‐years. We did not detect any differences when comparing participants using daily with participants using event‐driven PrEP.

**Table 1:** Incidence rate of STIs

STI	IR per 100PY	95% CI
Syphilis	8.26	5.49 to 12.42
*Neisseria gonorrhoeae*	38.94	31.78 to 47.72
Pharynx	16.18	12.12 to 21.59
Anal	24.78	19.43 to 31.60
Urine	5.90	3.71 to 9.36
*Chlamydia trachomatis* (non‐LGV)	30.92	24.73 to 38.65
Pharynx	4.54	2.69 to 7.67
Anal	18.72	14.23 to 24.63
Urine	10.20	7.13 to 14.59
*Chlamydia trachomatis* (LGV)	4.22	2.45 to 7.27
Pharynx	0.00	NA
Anal	4.22	2.45 to 7.27
Urine	0.00	NA
*Chlamydia trachomatis *(LGV + nonLGV)	34.21	27.59 to 42.42
*Mycoplasma genitalium*	21.35	16.09 to 28.33
Pharynx	3.50	1.82 to 6.72
Anal	14.24	10.17 to 19.93
Urine	4.73	2.69 to 8.33
Bacterial STI	75.39	63.81 to 89.08
*Trichomonas vaginalis*	0.95	0.31 to 2.95
Pharynx	0.00	NA
Anal	0.63	0.16 to 2.53
Urine	0.32	0.04 to 2.24
Hepatitis C	2.93	1.53 to 5.64
Hepatitis B	0.00	NA
HSV‐2	8.13	4.98 to 13.27
HIV	0.00	NA
Any STI	84.11	71.47 to 98.97

**Figure 5:**
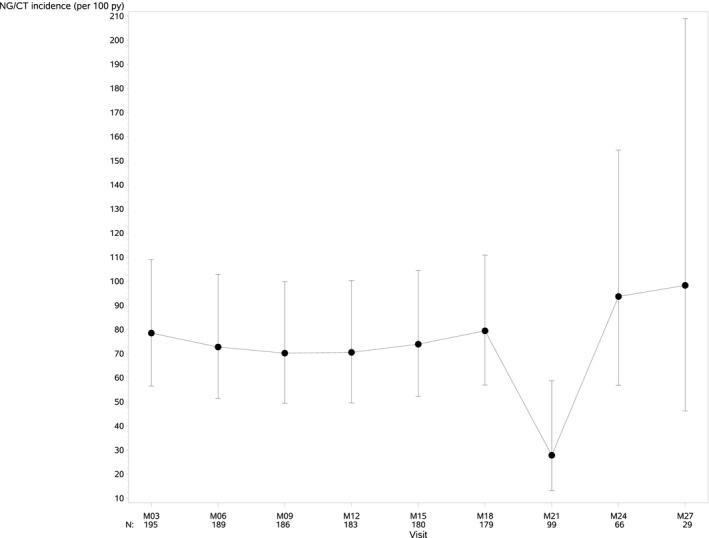
Trend of NG/CT tri‐monthly incidence.

We did not detect a significant change of NG/CT incidence over time (Poisson mixed effects model, *p* = 0.38) (Figure 5).

## 4 Discussion

This study showed that 200 MTSM at high risk for HIV in Belgium adopted both daily and event‐driven PrEP as an HIV prevention method tailored to their sexual life style.

Be‐PrEP‐ared was one of the first European PrEP demonstration projects among MTSM at high risk for HIV which allowed for choosing between daily or event‐driven PrEP, and for switching regimens according to users’ preferences. Our results showed a substantial proportion of PrEP users switching regimens during follow‐up (19%), which is an indication of users’ varying needs and preferences over time. A similar study in Amsterdam found a cumulative proportion of 30% of participants who had switched regimens two years after initiation of PrEP 23. In addition, 32.0% of our study participants interrupted using PrEP at least once, for various reasons. These results suggest that in real life, “daily” and “event‐driven” PrEP may be fluid categories rather than rigid regimens. Rather than proposing daily or event‐driven PrEP, it may be more useful to propose PrEP as a daily regimen, which can be interrupted and adapted to short or long episodes of individual protection needs. Given the importance of adherence, emphasis in patient education and counselling should thus be given on how to safely start and stop PrEP episodes rather than focusing on following a particular regimen.

Event‐driven PrEP use may lead to lower adherence compared to daily use, as suggested by other studies 13, 24. In our study, we found a proportion of covered sex days of 67% while on event‐driven and 96% while on daily PrEP. In the Adapt study, the proportion covered sex acts was 74% and 85% among MSM in Bangkok and 52% and 75% among women in Capetown, for event‐driven and daily PrEP respectively 13, 24. It should be noted however that participants of the Adapt study were randomly assigned to a PrEP regimen, as opposed to our study, where participants self‐selected and could switch between daily and event‐driven, according their preference, during the entire follow‐up period. In the Ipergay Open Label study in France, where participants only had the option of event‐driven PrEP, 50% correct doses were taken. The results are hard to compare not only because of differences in study design but also because none of the previous studies reported on the coverage of sex acts in relation to risk exposure. As indicated before, being able to safely use PrEP when it is needed may be of paramount importance, rather than focusing on rigidly following a particular dosing regimen for indefinite period of time. Consistent high adherence during periods of non‐exposure is costly 11. Therefore, Haberer et al. called for a novel paradigm: prevention‐effective adherence that proposes the use of PrEP only during periods of heightened risk exposure resulting into effective protection against HIV acquisition 25. It also includes the use of other effective HIV prevention tools such as condoms and other behavioural strategies 25. Risk‐taking can be seasonal and fluctuations are determined by personal and contextual factors 26. PrEP adherence will thus depend on users’ ability to understand their risk for HIV acquisition, and act upon it. When measuring adherence in that sense (i.e. the proportion of high risk sex acts covered by PrEP), our study participants were highly adherent to PrEP. Adherence was low for men using event‐driven PrEP in low exposure situations, e.g. sex with the steady partner, or sex protected by a condom. Low adherence in low‐risk situations may be of lesser concern and it fits into an effective “prevention‐effective adherence” strategy.

Our results show some interesting changes in participants’ sexual behaviour: while numbers of casual and anonymous sex partners did not increase, the frequency of condom use with these partners dropped significantly during follow‐up. Anonymous partners are usually connected with very short‐term sexual interactions and few negotiation of using protection strategies 27. Our results compare well with results of the Amsterdam PrEP cohort study. In this study, the number of condomless anal sex acts with casual partners increased over time, whereas the number of partners and sex acts were stable 28. Also in France, the rate of condomless sex at last intercourse increased from 53.3% at baseline to 79% at month 12 29. In the light of prevention‐effective adherence, the terminology of “risk taking” and hence its assessment may no longer be appropriate for HIV. The high efficacy of PrEP seen in demonstration projects and in the real world suggests that “risk compensation” does not necessarily result in an increased risk for HIV infection in PrEP users 30.

In contrast to zero new HIV infections, we found a relative high incidence of STI during follow‐up, specifically for NG and CT (incidence rate of NG and CT respectively 38.9 and 34.2 per 100 person‐years).

We did not find an increasing trend when analysing three‐monthly incidences of NG/CT over time. These incidences corroborate other PrEP demonstration studies’ results 31. Neither the PROUD trial nor the Ipergay trial reported increased STI frequency during study follow‐up 1, 4. Since our study did not include a control group of highly exposed non‐users, we cannot conclude that PrEP use does not increase STI incidence. STI incidence data before PrEP use were also not available. A study in Canada evaluated the impact of PrEP on STI in a cohort of MSM before and after initiation of PrEP, and observed a 72% increase in incidence of STIs in the 12 months after PrEP 32. In a similar study in Australia STI incidence increase from 69.5 prior to PrEP to 98.4 per 100 person‐years during PrEP 33. The effect of PrEP use on STI incidences remain uncertain and should further be studied. The incidence of hepatitis C among HIV‐negative participants was higher than expected in our study, as also observed in the Amsterdam PrEP cohort 34. These results call for regular screening for STI, including hepatitis C, as an essential component of PrEP delivery.

Some limitations and potential biases should be acknowledged: The original study follow‐up duration of 18 months was extended a few months for a limited number of study participants. As the inclusion order in the study was random, a selection bias due to the extension may not apply. However, the number of participants at month 21, 24 and 27 was very small, decreasing the precision of our study results after month 18.

Although reporting bias cannot be excluded, the use of digital tools as opposed to face‐to‐face interviews may have reduced this bias 35.

The online diary had its limitations to avoid burdening study participants too much with detailed data provision. For instance, it did not include the timing of sexual intercourse, preventing us from adjusting adherence by the correct timing of the PrEP intake. Furthermore, some participants did not fill it in regularly, or filled it retrospectively after some days, which may have led to a recall bias.

## 5 Conclusions

In conclusion, PrEP is an effective, safe and well adopted HIV prevention tool for MTSM who are at high risk for HIV in Belgium. Participants adapted daily and event‐driven regimens to their own needs and were able to adapt their PrEP adherence to risk exposure. Monitoring of PrEP use in Belgium is needed to confirm these findings among regular PrEP users outside of the context of a demonstration project.

## Competing interests

Gilead Sciences donated the study drug (Truvada). No other competing interests.

## Authors’ contributions

BV contributed to conception of the protocol, writing of the first draft of the manuscript, revision and editing of the present version of the manuscript. TR contributed to conception of the protocol, writing of the first draft of the manuscript and revision of the present version of the manuscript. IDB contributed to conception and writing of the protocol focussing on laboratory methods, writing of the first draft of the manuscript and revision of the present version of the manuscript. CN contributed to conception of the protocol focussing on behavioural sciences, writing of the first draft of the manuscript and revision of the present version of the manuscript. TC contributed to conception and writing of the protocol, supervision of laboratory methods, writing of the first draft of the manuscript and revision of the present version of the manuscript. JB contributed to conception and writing of the protocol focussing on statistical methods, data analysis and reporting, writing of the first draft of the manuscript and revision of the present version of the manuscript. CK and KW contributed to conception of the protocol, writing of the first draft of the manuscript and revision of the present version of the manuscript. ML contributed to global supervision of the protocol writing, writing of the first draft of the manuscript and revision of the present version of the manuscript.

## Abbreviations

ALT/AST, alanine transaminase/aspartate transaminase; ARV, antiretroviral; CT, chlamydia trachomatis; eCRF, electronic Case Report Form; FTC, emtricitabine; FU, follow‐upx0020; HBV, hepatitis B virus; HCV, hepatitis C virus; HIV, human immunodeficiency virus; HSV‐2, herpes simplex‐2 virus; IRB, Institutional Review Board; ITM, Institute of Tropical Medicine; MSM, men who have sex with men; MSTM, men and transgender women who have sex with men; MG, Mycoplasma genitalium; NG, Neisseria gonorrhoeae; PrEP, pre‐exposure prophylaxis; (S)AE, (serious) adverse event; STI, sexually transmitted infections; TDF, tenofovir disoproxil fumarate; TV, Trichomonas vaginalis; TFV, tenofovir; TFV‐DP, tenofovir diphosphate.

## Funding

The Applied Biomedical Research (TBM) Program of the Belgium Research Agency (IWT).
